# Parental stress and child stimulation practices: examining associations with child developmental outcomes over time in Kenya and Zambia

**DOI:** 10.1186/s40359-024-01533-y

**Published:** 2024-01-26

**Authors:** Kenneth Okelo, Aja Louise Murray, Josiah King, Patricia Kitsao-Wekulo, Silas Onyango, Margaret Nampijja, Bonnie Auyeung

**Affiliations:** 1 Department of Psychology, School of Philosophy, Psychology and Language Sciences, University of Edinburgh, 7 George Square, EH8 9JZ Edinburgh, UK; 2 African Population and Health Research Center, Nairobi, Kenya

**Keywords:** Child development, Parental stress, Child stimulation practices and childcare

## Abstract

**Background:**

Parental stress often arises when parenting demands exceed the expected and actual resources available for parents to succeed in the parenting role. Parental stress is an important contributor to parent-child relationships. This, in turn, affects opportunities to engage their children in stimulating activities which could improve their development outcomes. However, limited evidence exists from sub-Saharan Africa (SSA) on the association between parental stress, caregiving practices, and child developmental outcomes.

**Methods:**

The findings reported in this paper were derived from data collected through previous longitudinal work on nurturing care evaluation studies in Kisumu and Nairobi Counties in Kenya, and Chisamba District in Zambia. A total of 341 caregivers and their children who participated in the three rounds of data collection were included in this study. The children’s mean age was 9.3 (SD = 8.2) months pre-intervention, 25.5 (SD = 8.6) months in mid-intervention, and 36 (SD = 10.0) months post-intervention. The Ages and Stages Questionnaire (ASQ), Parental Stress Scale (PSS), and caregiving tools were used to assess children’s developmental outcomes, parental stress, and stimulation practices, respectively. A Random Intercept Cross-Lagged Panel model (RI-CLPM) was used to determine the association between caregivers’ parenting stress, child stimulation practices, and child developmental outcomes.

**Results:**

The findings showed that caregiver stimulation practices were positively associated with developmental outcomes. Findings on the associations between parental stress and caregivers’ stimulation practices and children’s developmental outcomes were not universally supported.

**Conclusion:**

The findings show that improved caregiver stimulation practices are likely to improve children’s developmental outcomes. The policy implications of the findings from this study focus on improving parenting practices by addressing the predictors of parental stress. This includes subsidising childcare services to reduce costs.

**Trial registration:**

Pan African Clinical Trials Registry (https://pactr.samrc.ac.za/) database (ID number: PACTR20180774832663 Date: 26/July/2018.

## Introduction

Parental stress, which often arises when parenting demands exceed the expected and actual resources available for parents to succeed in the parenting role [1], negatively affects parent-child relationships [2, 3]. Therefore, exposure to parental stress during pregnancy and the postnatal period can result in delayed developmental outcomes. Studies have also linked parental stress during pregnancy with an increased risk of premature birth, schizophrenia, and low IQ in the offspring [4, 5]. Similar associations have been reported in previous genomic studies. That is, adversities related to stress have been associated with epigenetic patterns in neonates [6]. Studies examining maternal stress in pregnant women have shown an association between parental stress and transfer (methylation) of the *CpG* site of the *NR3C1* promoter in the cord blood of newborns [7]. Replicated studies have also reported increased NR3C1 DNA methylation in male infants among parents with depression symptoms [8]. *NR3C1* is a glucocorticoid (GC) receptor gene, exposure to early-life stressors can alter/result in a life-long increase in GC secretion and may result in disruption of the homeostatic mechanisms regulating hypothalamic-pituitary-adrenal (HPA) axis [9]. Therefore, this predisposes children to the risk of developing stress-related diseases such as anxiety disorders, borderline personality disorder (BPD), mood and affective disorders, and posttraumatic stress disorder (PTSD) [9, 10]. Although there are numerous studies on DNA methylation in child development, questions about its reliability and validity remain, such as understanding stable markers and the period of their stability hinders the replicability of such studies [11].

Notably, parental stress during postnatal has been linked to behavioural problems in children and dysfunctional parenting behaviours [12, 13]. In addition, studies have associated parental stress with parental burnout (physical, mental, and emotional exhaustion) and sleep disorders (disturbed or shortened sleep) [14, 15]. Parental burnout has negative effects on parents’ mental health and overall quality of life which could potentially affect their interactions with their child. A secure parent-child attachment/relationship is a central characteristic of responsive caregiving and child stimulation activities [16]. This implies that caregivers with secure attachments are more sensitive and responsive to their children’s needs and frequently engage their children in stimulation activities. They can modify their instructions/interactions appropriately in reaction to their children’s behaviour. Through this, the caregiver intentionally interacts with the child by carrying out stimulation activities aimed at improving their children’s developmental outcomes.

Regular engagement of children in stimulating/play activities is reported to significantly promote children’s developmental outcomes including fewer behavioural problems, higher intelligence scores, and positive academic outcomes [17]. In addition, play and stimulation activities significantly improve children’s cognitive abilities [18]. This is often observed in a transactional approach where engagement in stimulating activities also increases a parent’s cognitive abilities in subsequent parenting [19]. That is, frequent engagement in play/stimulating activities improves caregiving knowledge and skills, leading them to practice positive parenting in the future.

**Fig. 1:**
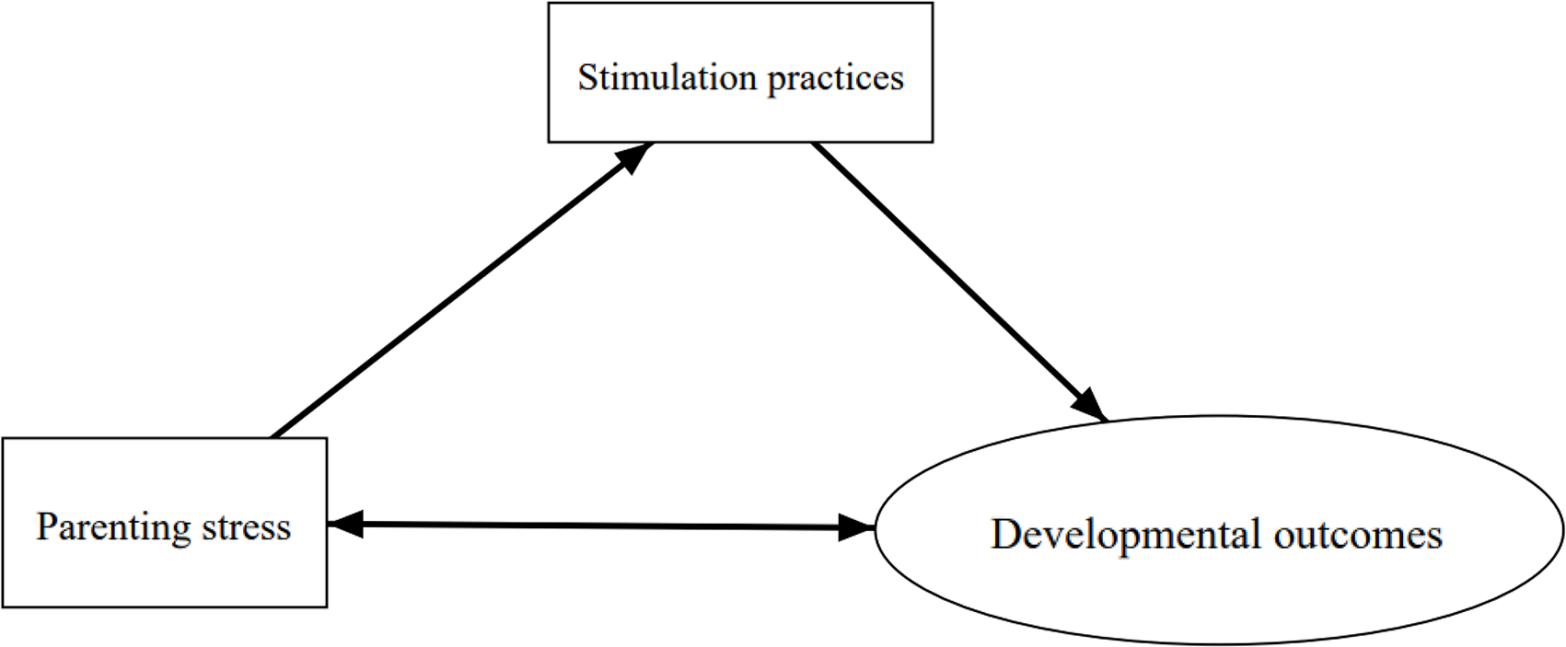
Conceptual framework on the relationship between parental stress, caregiving practices and developmental outcomes (source; own)

It is estimated that 250 million children aged less than five years in low- and middle-income countries (LMICs) are at risk of delayed developmental milestones [20]. Over 66% live in sub-Saharan Africa (SSA) and are at risk due to factors such as poor nutrition, family poverty, high Human Immunodeficiency Virus (HIV) prevalence, and under-stimulation in the home environment [21, 22]. Noting that parental stress has been linked to poverty which is salient in SSA, the level of parental stress in low-resource settings in SSA might be higher [23–27]. Limited studies have been done on the contributions of parental stress on child stimulation in sub-Saharan Africa (SSA). Therefore, this study sought to understand the relationship between parental stress, parenting stimulation practices, and children’s developmental outcomes in disadvantaged settings in SSA, as shown in the conceptual framework in Fig. 1.

This study aimed to use a dataset from longitudinal nurturing care evaluation studies conducted in Kisumu County in Kenya and the Chisamba District in Zambia [28] to investigate the relationship between parental stress, parenting stimulation practices, and children’s developmental outcomes over time.

The hypotheses of this study were as follows: (a) low parental stress scores are associated with frequent child stimulation practices, (b) low parental stress scores are associated with higher child developmental outcome scores, (c) frequent child stimulation practices are associated with higher child developmental outcome scores, and b) there is a reciprocal association between parental stress, stimulation practices, and child developmental outcomes.

## Methodology

### Study design

The study reported in this paper was derived from data collected through earlier longitudinal nurturing care evaluation studies conducted in Kisumu County in Kenya and Chisamba District in Zambia [28]. In these two studies, caregiver-child dyads were assigned to either the intervention arm (to receive nurturing care intervention) or the control arm (to receive standard care provided by the Ministry of Health and Ministry of Education in the respective countries). The caregiver-child dyads were drawn from villages/clusters that were purposively selected to ensure a buffer zone between the intervention and the control arms. The intervention was implemented by the Episcopal Relief & Development (ERD) team, together with the Zambia Anglican Council Programmes (ZACOP) in Zambia and with ACK Development Services (ADS) Nyanza in Kenya. The nurturing care intervention program was designed to enhance children’s cognitive, language, motor, social, and emotional development, as well as promote positive discipline and parenting overall. The program was implemented through trained ECD promoters, who facilitated sessions on positive parenting through home visits and support and learning group meetings [29]. The program had an intensive 24-month parental participation timeframe (24 group meetings and 24 ECD home visits). In addition, the project utilised the rich church structure and its wide reach for ECD program delivery to community leaders during weekly Sunday services, faith leaders’ meetings, pastoral visits to households, and cell group meetings. The intervention targeted children aged below three years.

### Study sites

This study was conducted in Kenya and Zambia. In Kenya, the research took place in Kisumu County, specifically in Awasi-Onjiko, a sub-location within one ward of the Nyando sub-county. Kisumu County has a population of 1,131,982 individuals, with a growth rate of 2.6%, and is divided into six administrative Sub counties [30]. The county has one provincial hospital, two sub-county hospitals, 16 public health centres, 27 public dispensaries and five private hospitals. There were also four nursing homes and five private dispensaries. The average distance to a health facility is approximately six kilometres, and 67% of the population can access one within five kilometres. However, there are disparities in distance to the nearest health facility. The doctor-to-population ratio is 1:44,634, and the nurse-to-population ratio is 1:2,383. Although antenatal care attendance was relatively high at 71%, most mothers (54%) delivered at home. The proportion of women using contraceptives is low, estimated at 27%, compared to the national average of 46% [30]. The Nyando sub-county comprises five wards, namely, Awasi/Onjiko, Ahero, Kabonyo/Kanyagwal, Kobura, and East Kano/Wawidhi, with a total population of 141,037. Of this population, females account for approximately 49% [30]. The Ministry of Health (MOH) Health Information System (HIS) identified Ayucha, Boda 1, and Wanga’ng’a in Onjiko/Awasi Ward as the most vulnerable areas in the entire Nyando sub-county and recommended implementing nurturing care interventions at these sites.

In Zambia, the study was conducted in the Mwantaya and Chamuka wards, situated in Chisamba District in Central Province. The population of Chisamba district in 2010 was 103,983, and it had a higher HIV prevalence rate than the national average in rural Zambia (13.4%) [31]. Moreover, malnutrition rates were also high, with 42.1% of children under the age of five exhibiting stunted growth. Only 46.5% of the mothers had skilled professionals attending their deliveries, and fewer than a quarter (14.4%) of the population lacked formal education. According to 2010 data, Chamuka Ward had a population of 21,210, with 10,685 males and 10,525 females living in 3833 households. Mwantaya Ward had very little infrastructure and only one health clinic, despite being sparsely populated [31].

### Participants

In rural Zambia, the survey design was a cluster-randomised controlled trial, in which the cluster was the community. The study team followed Hemming and Girling’s [32] study for sample size calculation by fixing the number of clusters to at least five in each arm. Hence, they assumed that the intervention could yield an effect size of 0.4 in terms of ECD parenting practices with an intracluster correlation (ICC) of = 0.03. The team also estimated a confidence interval of 95%, a margin of error of 5%, a power of 80%, and an attrition rate of 10%. Thus, the total sample size for each arm was 255 (510 primary caregivers in total). However, only 395 primary caregivers met the inclusion criteria; children aged below 18 months or who were pregnant and in their third trimester were identified and recruited from the households. Of the 395 caregivers recruited, 176 participated in the three rounds of data collection (pre-intervention, mid-intervention, and post-intervention).

**Fig. 2:**
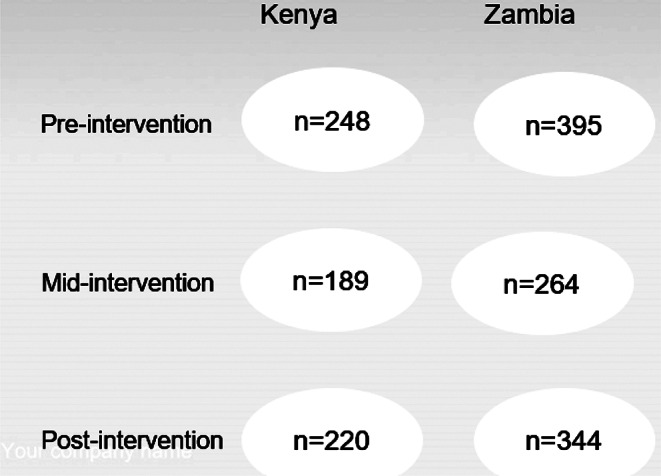
The flow of study participants

In rural Kenya, the survey was a cluster quasi-experiment, in which the cluster was a village nested in a ward. The study team followed Hemming and Girling’s [32] study for sample size calculation by fixing the number of clusters to at least five in each arm. There were two project implementation sites: three clusters at each site. Due to limited resources, with six clusters in each arm, the ICC was set at 0.02, the effect size at 0.43, and the dropout rate at 10%. With this information, the total number of caregivers in each arm was 129, and the cluster size was 21.5 caregivers. Due to rounding issues, the cluster size was 22 caregivers and the number of caregivers per arm was 132 caregivers, implying a total sample size of 264 for the two arms. Of the 264 caregivers recruited, 165 were included in the analysis. They participated in three rounds of data collection and were eligible for the collection of the outcome and predictor variables. The outcome variable was collected from children aged one month to 60 months; therefore, only children aged between 1 month and 36 months pre-intervention were included in this study. The total number of participants from the two study sites who were included in this study was 341 (Fig. 2).

### Measures

Using questionnaires, information on caregivers’ socio-demographics, caregiving knowledge, attitudes and practices, and health-seeking behaviour was collected. The parental stress scale and the Ages and Stages Questionnaire (ASQ) were used to assess parental stress and child development, respectively. The predictors (PSS and stimulation practices) and outcomes (ASQ) were included in the primary caregiver questionnaire. The questionnaire was administered by trained field interviewers and the average duration of each interview was estimated to be one hour. Interviews and child assessments were conducted at caregivers’ homes. Quality control was ensured through spot checks, supervision, and weekly team debriefs.

### Outcomes

The main outcome of this study was child development. These included gross and fine motor skills and language, socio-emotional, and cognitive development measured using the Ages and Stages Questionnaire– Third Edition (ASQ-3) [33]. This was done through a combination of primary caregiver self-reported questions and direct observations by trained field interviewers, similar to the procedures used in a study conducted in South Africa and Zambia [34]. Apart from self-reports, primary caregivers were requested to try each activity with their children to facilitate an accurate item assessment. Items were scored ‘yes’ (= 10 points) if the child was able to perform the activity, ‘sometimes’ (= 5 points) if the child tried and failed but the primary caregiver reported that the child could perform the activity sometimes, and ‘no’ (= 0 points) if the child was unable to perform the item. The responses to each domain’s six questions were summed to obtain a score for each area. The scores for each domain range from 0 to 60. Higher scores indicated outcomes that were more positive for children. To calculate the total ASQ score, the total score in each domain was summed, and the total score ranged from 0 to 300. The internal consistency of the ASQ tool was determined for the total score by using Cronbach’s alpha, and the result was 0.79.

### Predictors

The first predictor was the caregiver’s parental stress level. The Parenting Stress Scale (PSS) has 18 questions that assess the level of parenting stress [35]. The PSS tool has been previously used in South Africa [36], and studies on its validity and reliability have indicated good internal consistency, construct validity, convergent validity, and test-retest reliability [35, 37–41]. In this study, the PSS tool was translated into the local languages of Dholuo in Kenya, Nyanja, and Tonga Zambia. These questions were asked before the intervention (pre-intervention), one year into the intervention (mid-intervention), and at the end of the intervention (the intervention period was two years). The PSS tool was used to obtain information from parents about their feelings and perceptions of their parenting experiences. In each dataset, the caregiver’s PSS responses were assigned scores (*5-point Likert Scale; 1 = Strongly Disagree, 2 = Disagree, 3 = Not Sure, 4 = Agree, 5 = Strongly Agree*). To compute the parental stress score, items 1, 2, 5, 6, 7, 8, 17, and 18 which were positively worded were reversed and scored as follows: (1 = 5) (2 = 4) (3 = 3) (4 = 2) (5 = 1). The item scores were then summed up. A low score signified a low level of stress, whereas a high score indicated a high level of stress. The overall possible scores on the scale ranged from 18 to 90.

Caregivers’ play and stimulation practices were the second predictors. A structured questionnaire was used to collect information on the caregivers’ parenting practices in each developmental domain. The questionnaire was adopted from nurturing care intervention program activities [29]. They were asked a set of questions to establish engagement in the child’s stimulation in the previous week in each developmental domain (cognitive, language, motor skills, social, and emotional development). Their practices were reported and scored as follows: Yes = 1 and No = 0. The total expected score (highest score) in each domain was cognitive = 6; language = 6; motor skills = 4; social = 5; and emotional development = 5. To calculate the total practice scores, the total score in each domain was summed, and the total score ranged from 0 to 26.

### Statistical analysis

The data were cleaned and analysed using R software and R Studio [42]. Our analysis commenced with descriptive statistics of participants’ demographic characteristics. We also assessed the internal consistency of the ASQ, PSS, and stimulation practice questionnaire by using Cronbach’s alpha. Our findings revealed acceptable internal consistency for the total ASQ (0.79), total PSS (0.79), and total practice score (0.77) [43].

We then explored the association between parental stress, caregivers’ practices, and children’s developmental outcomes at the three-time points using an extension of a Random Intercept Cross-Lagged Panel Model (RI-CLPM) in R [44]. The RI-CLPM regression model was used to explore the association between two or more variables measured repeatedly over time. Using the RI-CLPM regression model, we were able to estimate within- and between-person effects as well as adjust for between-person covariates, such as demographic characteristics and intervention [45, 46]. The model allowed for the investigation of time-lagged associations between parenting stress, practices and child developmental outcomes. In addition, the model allowed us to explore reciprocity in the association between parental stress, stimulation practices, and child development at each time point. Further, we conducted a child developmental domain-specific model (personal social, problem-solving, communication, gross motor, and gross motor) as outcome variables and PSS and stimulation practices and predictors. This enabled us to check whether the association between PSS, stimulation practices, and child developmental outcomes was domain-specific. Our sensitivity analysis was performed using a multi-group model to assess whether there was a major difference between the results for participants in the intervention arm. The models were fitted using *Lavaan.survey* to account for the clustered structure of the data. The results showed no major differences between the two groups. Full analysis code is provided at OSF. To assess the model fit, we used the Comparative Fit Index (CFI), Tucker–Lewis’s index (TLI), Standardised Root Mean Square Residual (SRMR), and Root Mean Square Error of Approximation (RMSEA). Good fit was judged based on values greater than or equal to 0.95 for CFI and TLI, and less than or equal to 0.06 for SRMR and RMSEA [47].

### Ethical considerations

Permission to use the current datasets was sought from the African Population and Health Research Centre (APHRC). Among the studies that collected the data, ethical approval was obtained from institutional review boards (IRBs) in Kenya and Zambia to conduct the research in their respective countries. Written informed consent was obtained from the study participants (parents/guardians) before data were collected. For respondents who could neither read nor write, a witnessed thumbprint was used to sign a consent form. Consent was obtained at every round of data collection. Consent documents and questionnaires were translated into Dholuo, the local dialect of the Kenyan rural study site, and Nyanja and Tonga, the local dialects of the Zambia rural study site. Confidentiality of the data and the participants’ privacy were observed during and after data collection. These rural studies were registered under the trial registration number PACTR20180774832663.

## Results

### Demographic characteristics

All participants in this study were female primary caregivers. That is biological mothers or those aged above 18 years whose primary responsibility was taking care of the child. Their demographic characteristics remained stable across the phases of this study, with a slight upward trajectory in the proportion of those who reported being married, ages ranging from 20 to 39 years, education above primary level, and with more than one child. Notably, there was a significant increase in the proportion of caregivers who reported earnings between USD 76 and USD 100 per month at both study sites. On the children’s demographic, slightly above the average were females (male; 48.4% and 51.6%), the children's mean age was 9 months at pre-intervention, 21 months at mid-intervention and 33 months at post-intervention as shown in Table 1).

**Table 1:** Comparison of mean children ASQ scores, parental stress scores, stimulation practices scores with demographic characteristics at baseline

	Pre-intervention-Zambia				Pre-intervention - Kenya			
	*N* = 176(%)	ASQ scores Mean (SD)	PSS scores Mean (SD)	Practices scores Mean (SD)	*N* = 165(%)	ASQ scores Mean (SD)	PSS scores Mean (SD)	Practices scores Mean (SD)
Age of the primary caregiver								
Below 20 years	28 (15.9)	183.2 (108.6)	45.9 (9.0)	12.0 (8.0)	15 (9.1)	166.4 (102.3)	43.9 (15.0)	13.0 (7.1)
20 and 29years	85 (48.3)	148.1 (105.1)	44.2 (8.5)	11.6 (7.8)	80 (48.5)	151.1(118.9)	39.8 (14.7)	10.1 (7.4)
30 to 39 years	52 (29.5)	165.5 (108.2)	40.6 (9.6)	12.8 (8.2)	57 (34.5)	164.3 (115.5)	37.5 (9.7)	13.0 (6.9)
40 and above years	11 (6.2)	220.0 (87.2)	40.5 (10.3)	15.8 (8.2)	13 (7.9)	197.0 (94.1)	40.0 (10.63)	13.0 (5.7)
Marital status								
Single parent	54 (30.7)	165.9 (105.1)	44.4 (8.3)	12.9 (8.7)	31 (18.8)	171.0 (111.2)	37.7 (10.9)	12.8 (6.8)
Married	122 (69.3)	162.1 (107.6)	42.7 (9.6)	12.0 (7.6)	134 (81.2)	190.2 (93.4)	45.0 (9.8)	12.4 (5.7)
Education								
Primary and below	106 (60.2)	157.0 (109.5)	42.3 (9.0)	11.8 (8.3)	65 (39.4)	130 (128.6)	34 (21.6)	8.2 (8.3)
Secondary	70 (39.8.2)	172.9 (101.8)	44.6 (9.5)	12.9 (7.5)	98 (59.4)	182 (94.6)	41.4 (9.6)	12.7 (5.5)
					2 (1.2)	171.4 (116.9)	37.6 (11.2)	13.0 (7.2)
Income								
Below USD 50	14 (8.0)	173.6 (101.1)	43.6 (8.6)	16.3 (6.4)	48 (29.1)	172.0(113.2)	35.2(12.5)	12.6(7.1)
Between USD 50–75	15 (8.5)	158.7 (94.9)	41.2 (7.7)	10.7 (8.6)	26 (15.8)	186.0 (96.4)	48.11(8.4)	11.9 (5.6)
Between USD 76– 100	145 (82.4)	162.6 (109.2)	43.4 (9.5)	12.2 (8.0)	91(55.2)	172.80 (109.4)	38.6 (9.5)	13.0 (6.6)
Above USD 100	2 (1.1)	175 (77.8)	41.5 (0.7)	3.5 (2.1)	0(0)	NA	NA	NA
Number of children below three years								
1	147 (83.52)	164.7 (107.2)	43.2 (9.0)	12.5 (8.1)	128 (77.6)	167.8 (110.7)	38.6 (11.3)	12.5 (6.9)
2	28 (15.9)	151.1 (102.7)	42.6 (10.4)	10.4 (7.0)	34 (20.6)	194.1 (98.8)	41.0 (10.3)	13.7(5.2)
3 and above	1 (0.6)	300 (NA)	51 (NA)	25 (NA)	3 (1.8)	245 (43.38)	41.3 (12.7)	11.7 (2.9)

### Parental stress score, stimulation activities and children’s developmental scores

**Fig. 3:**
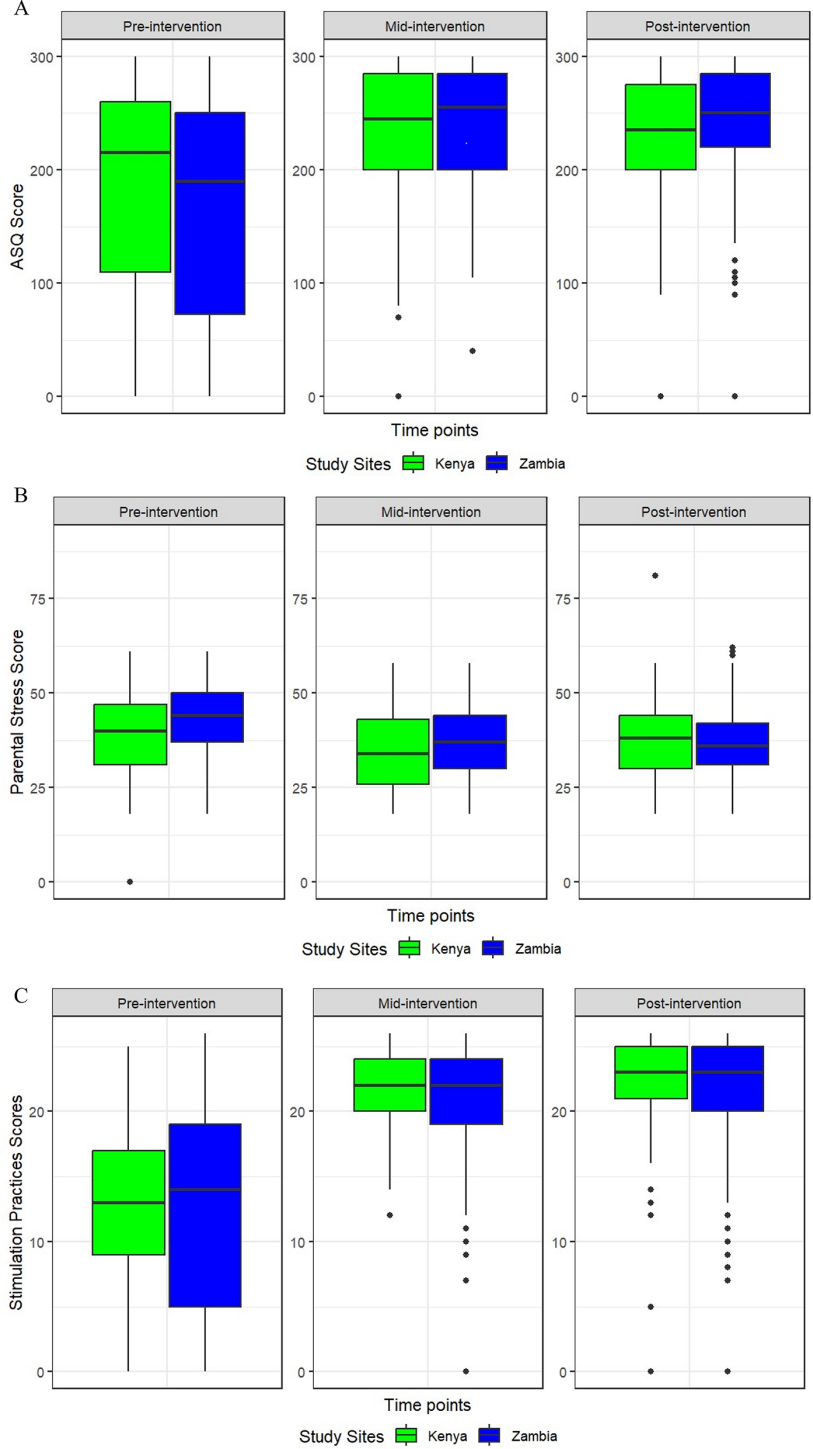
(**A**) ASQ scores by round compared by study sites. (**B**) Parental stress scores by round compared by study sites. (**C**) Stimulation practice scores by round compared by study sites

From the findings, the mean total ASQ score increased from pre-intervention to post-intervention for Zambia study sites (pre-intervention; 163.3 (SD = 106.5), mid-intervention; 240.0 (SD = 53.2) and post-intervention; 242.2 (SD = 51.1)). In Kenya, the mean total ASQ score increased from pre-intervention to mid-intervention and then decreased slightly from mid-intervention to post-intervention (pre-intervention: 174 (SD = 108.1), mid-intervention; 236.7 (SD = 55.6), and post-intervention; 221.0 (SD = 73.8)). However, the mean total ASQ score was higher for Zambia than for Kenya at two-time points (mid- and post-intervention), and the difference between the sites was larger post-intervention than pre-intervention. Regarding the stimulation practices, a similar trend was observed with an increase from pre-intervention to post-intervention in the Zambia study site (pre-intervention; 12.3 (SD = 8.0), mid-intervention; 20.8 (SD = 5.1), and post-intervention; 21.3 (SD = 5.8)) and a slight decrease from mid-intervention to post-intervention in the Kenya study site (pre-intervention; 12.7 (SD = 6.6), mid-intervention; 21.7 (SD = 3.2), and post-intervention; 21.3 (SD = 6.2)). This trend was also observed in PSS scores in Zambia (pre-intervention; 43.2 (SD = 9.2), mid-intervention; 37.0 (SD = 9.8) and post-intervention; 36.5 (SD = 9.1)) while in Kenya (pre-intervention; 39.1 (SD = 11.1), mid-intervention; 35.0 (SD = 10.1) and post-intervention; 37.5 (SD = 10.2)) as shown in Fig. 3A, B and C.

### Association between parental stress, stimulation activities and child developmental outcomes

#### Combined study sites

**Fig. 4:**
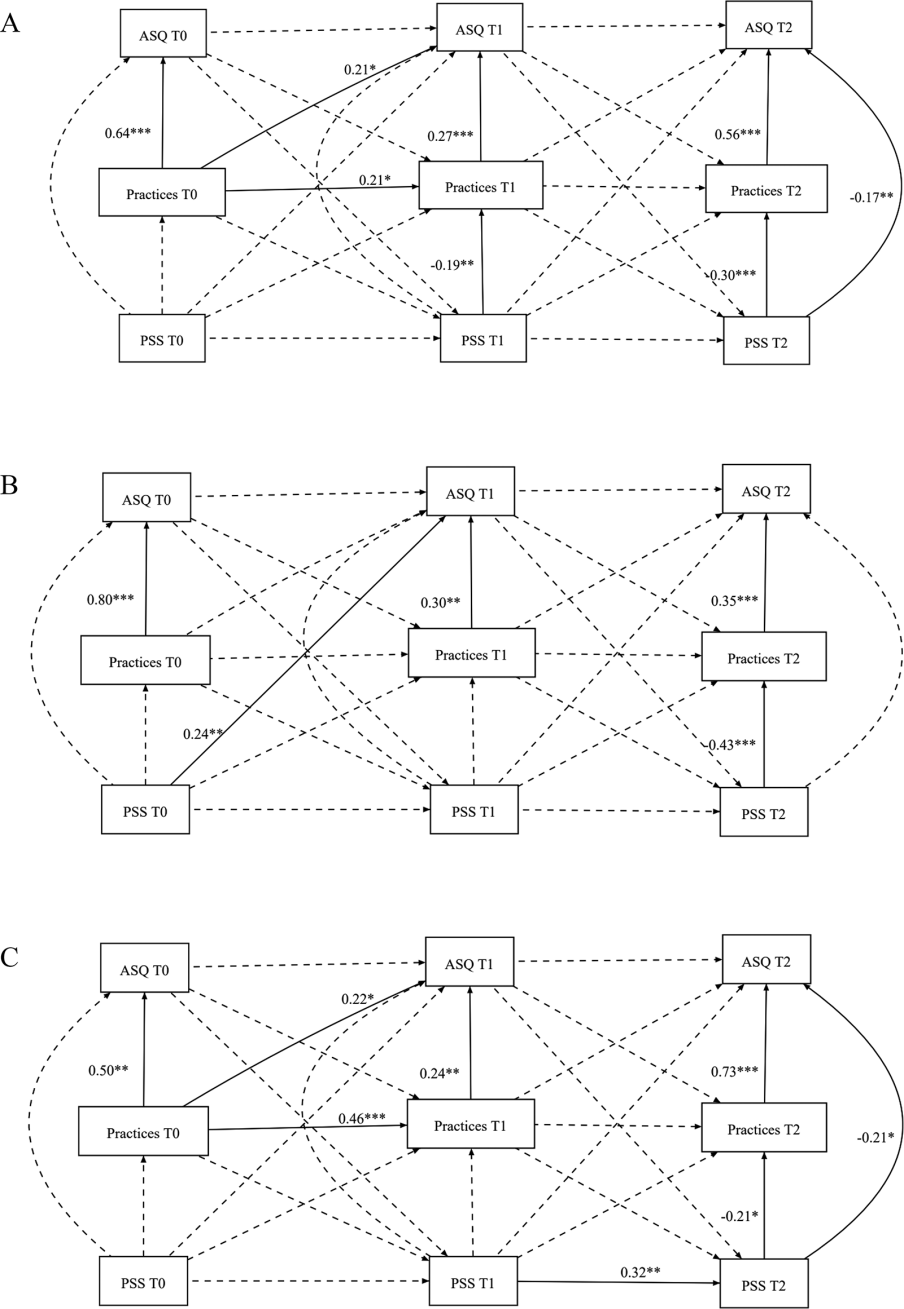
(**A**) Random intercept cross-lagged panel model of parental stress, stimulation practices, and ASQ– Combined study sites. (**B**) Random intercept cross-lagged panel model of Parental stress, stimulation practices, and ASQ– Zambia rural study sites. (**C**) Random intercept cross-lagged panel model of parental stress, stimulation practices, and ASQ– Kenya rural study sites. *Notes*: T0 = pre-intervention, T1 = mid-intervention, T2 = post-intervention. The broken lines denote insignificant paths

When fitting the extended Random Intercept Cross-Lagged Panel Model (RI-CLPM) on the combined study site, the results showed a good fit, with CFI, TLI, RMSEA, and SRMR values of 1, 0.99, 0.016, and 0.008, respectively. Regarding the association between stimulation practices and child developmental outcomes, the findings showed that primary caregivers of children with better ASQ scores at T1 had higher stimulation practices at T0 (T1; β = 0.21, *P =*.02^*^). The findings from this study also indicated that primary caregivers of children with better ASQ scores had higher stimulation practices (reciprocal association) at all study points (TO: β = 0.64, *P <*.01^***^; T1: β = 0.27, *P <*.01^***^; and T2: β = 0.56, *P <*.01^***^). The findings on parental stress showed that primary caregivers with higher PSS reported reduced stimulating practices at T1 and T2 (T1: β = -0.19, *P =*.01^**^ and T2; β = 0.30, *P <*.01^***^). In addition, primary caregivers of children with low ASQ scores reported higher PSS scores at T2 (β = -0.17, *P =*.01^**^). (Fig. 4A; Table 2).

**Table 2:** Results from Random intercept cross-lagged panel model of Parental stress, stimulation practices, and ASQ

	Combined study sites								Zambia rural								Kenya rural							
	*B*	*SE*	*P*	*β*	CFI	TLI	RMSEA	SRMR	*B*	*SE*	*P*	*β*	CFI	TLI	RMSEA	SRMR	*B*	*SE*	*P*	*β*	CFI	TLI	RMSEA	SRMR
PSS T1																								
					1.0	0.99	0.016	0.008					1	1.1	> 0.01	0.05					0.99	0.98	0.024	0.22
PSS T0	0.023	0.087	0.8	0.022					-0.155	0.103	0.1	-0.155					0.182	0.149	0.2	0.179				
Practices T0	-0.066	0.078	0.4	-0.73					-0.079	0.164	0.6	-0.082					-0.139	0.098	0.2	-0.161				
ASQ T0	0.044	0.085	0.4	0.049					0.199	0.159	0.2	0.210					-0.003	0.103	0.9	0.004				
Practices T1																								
PSS T0	-0.108	0.078	0.2	-0.098					-0.040	0.099	0.7	-0.039					-0.094	0.109	0.4	-0.047				
Practices T0	0.207	0.094	0.03*	0.133					-0.001	0.176	0.9	-0.001					0.464	0.101	> 0.01***	0.223				
ASQ T0	0.058	0.086	0.5	0.058					0.096	0.162	0.6	0.097					0.108	0.095	0.3	0.184				
ASQ T1																								
PSS T0	0.077	0.080	0.3	0.069					0.248	0.097	0.01**	0.235					-0.055	0.130	0.7	-0.047				
Practices T0	0.205	0.088	0.02*	0.205					0.329	0.174	**0.06.**	0.324					0.219	0.105	0.04*	0.223				
ASQ T0	-0.076	0.061	0.8	-0.067					-0.146	0.184	0.4	-0.146					0.181	0.127	0.2	0.184				
PSS T2																								
PSS T1	0.095	0.083	0.3	0.094					-0.050	0.106	0.6	-0.050					0.326	0.125	0.01**	0.321				
Practices T1	-0.045	0.068	0.5	-0.050					-0.069	0.095	0.5	-0.071					-0.007	0.089	0.9	0.008				
ASQ T1	0.029	0.067	0.7	0.031					0.128	0.094	0.2	0.135					-0.061	0.093	0.6	-0.069				
Practices T2																								
PSS T1	-0.082	0.079	0.3	-0.075					-0.087	0.101	0.4	-0.084					-0.083	0.127	0.5	-0.072				
Practices T1	-0.067	0.087	0.4	-0.067					-0.003	0.109	0.9	-0.003					-0.169	0.142	0.2	-0.170				
ASQ T1	-0.046	0.076	0.4	-0.046					-0.054	0.103	0.6	-0.055					-0.064	0.122	0.6	-0.063				
ASQ T2																								
PSS T1	0.011	0.077	0.9	0.010					0.172	0.098	0.08	0.164					-0.152	0.123	0.2	-0.135				
Practices T1	0.113	0.075	0.1	0.113					0.111	0.099	0.2	0.109					0.191	0.112	0.09	0.197				
ASQ T1	0.071	0.081	0.4	0.071					-0.058	0.105	0.6	-0.058					-0.097	0.127	0.4	-0.098				

Note that the PSS ranges from 18 to 90, ASQ scores from 0 to 300, and practice scores range from to 0–26. All variables were measured at three time points each (T0 at baseline, T1 after one year and T2 after two years).*Notes*: *Significant at *p <.05, **p <.01, ***p <.001.

### Zambia rural study site

When fitting the RI-CLPM model to the Zambian rural site, the results showed a good fit, with CFI, TLI, RMSEA, and SRMR values of 1.0, 1.1, 0.00, and 0.05, respectively. Regarding the association between stimulation practices and child developmental outcomes, the findings showed that primary caregivers of children with better ASQ scores at T1 had higher stimulation practices at T0 (T1; β = 0.32, *P =*.06). The findings from this study also indicated that primary caregivers of children with better ASQ scores had higher stimulation practices (reciprocal association) at all study points (TO: β = 0.80, *P <*.01^***^; T1: β = 0.30, *P =*.01^**^; and T2: β = 0.35, *P <*.01^***^). Regarding parental stress, the findings showed that primary caregivers with higher PSS reported reduced stimulating practice at T2 (T2: β = -0.43, *P <*.01^***^). In addition, primary caregivers of children with higher ASQ scores at T1 reported higher PSS scores at T0 (T1; β = 0.24, *P =*.01^**^), as shown in Fig. 4B; Table 2.

### Kenya rural study site

When the RI-CLPM model was fitted to the Kenyan rural site, the results showed acceptable CFI, TLI, RMSEA, and SRMR values of 0.99, 0.98, 0.024, and 0.022, respectively. The association between stimulation practices and child developmental outcomes, the findings show that primary caregivers of children with better ASQ scores at T1 had higher stimulation practices at T0 (T1; β = 0.22, *P =*.04^*^).

The findings from this study also indicated that primary caregivers of children with better ASQ scores had higher stimulation practices (reciprocal association) at all study points (TO: β = 0.50, *P =*.01^**^; T1: β = 0.24, *P =*.01^**^; and T2: β = 0.70, *P <*.01^***^). Regarding parental stress, primary caregivers with higher PSS reported low stimulating practice scores, and their children also reported low ASQ scores at T2 (Practices: β = 0.21, *P =*.03^*^ and ASQ; β = 0.22, *P =*.03^*^), as shown in Fig. 4C; Table 2.

We conducted an exploratory analysis to determine whether the association between parental stress and stimulation practices and child developmental outcomes was domain-specific. Our findings revealed a similar trend of mixed findings on the association between PSS and overall stimulation practice and total ASQ scores. In addition, the positive association between parental stress T0 and child development outcome T1 in the Zambia study site was only observed in the communication, personal social, and problem-solving domains (personal social; β = 0.26, *P =*.05^*^, communication; β = 0.31, *P =*.01^**^ and problem-solving; β = 0.24, *P =*.01^**^).

## Discussion

This study aimed to establish the association between parental stress, stimulation practices, and child developmental outcomes using datasets from longitudinal studies in two different African regions (rural Kenya and rural Zambia). The main findings of this study suggest a significant association between caregivers’ stimulation practices and children’s development outcomes. The association between parental stress and stimulation practices was observed only at the Kenya study site. Even though an association between parental stress and child developmental outcomes was observed in both countries, the positive association reported in Zambia was surprising.

Results from this study, though inconsistent across the study sites, show that parental stress was negatively associated with stimulation activities and child developmental outcomes. We speculate that this difference between study sites could be attributed to the different study designs used and other country-specific socioeconomic factors. These findings on the association between PSS and stimulation activities mirror those of other studies that have shown parental stress to be an inhibitor of caregivers’ participation in child-stimulating activities. As pointed out in other studies, excessive parental stress can induce negative and problematic interactions between parents and children, leading to dysfunctional parenting behaviour [48–50]. In studies among parents of preschoolers, parental stress was associated with dysfunctional parent-child interactions [49, 50]. Therefore, this affects opportunities for interaction and engagement of their children in stimulating activities. Similar findings were also observed in a meta-analysis in which parental stress was identified as a predictor of harsh parenting practices [51]. The moderate association observed in this study also conforms to other studies on parental stress and satisfaction [52].

Noting that early years form a critical period for children’s growth and development, engagement in stimulating activities promotes optimal growth and development. Studies focusing on early deprivation and institutionalisation of children have also supported early stimulation for optimal development across all domains [53]. Due to the high brain plasticity at this age, positive early experiences, such as play and stimulation, have a major influence on a child’s future cognitive, psychomotor, social-emotional and language development. In addition, developmental delays in early childhood might lead to long-term issues such as low academic and educational achievement, increased risk of criminal behaviour, and low income in adulthood. Such findings were also evident in the current study, with positive associations between caregivers’ stimulation practices and children’s ASQ scores, indicating that frequent engagement in stimulating activities can improve developmental outcomes. Therefore, children who frequently engaged in stimulating activities were more likely to have improved developmental outcomes.

However, the positive association observed between PSS scores at T0 and ASQ T1 at the Zambia rural study site was surprising. This implies that despite their stress level, such parents might have a higher awareness of the importance of engaging their children in stimulating activities and adequate resources to care for their children. Despite this, the negative associations demonstrated in this study, although not statistically significant, indicate that it is essential to address parental stress which has been identified as a risk factor associated with reduced stimulation practices and child developmental outcomes.

Strategies geared towards improving parental stress levels could be focused on addressing the background factors underlying parental stress, including poverty and low caregiver education [54]. On the other hand, to improve parent-child interaction and maximise opportunities for stimulation activities, global studies have shown that targeted interventions enhance mother-child interactions and increase developmental outcomes [55]. This systematic review documented evidence of such interventions in Bangladesh, China, India, and South Africa which were implemented through home visits, individual parent counselling sessions delivered through health facilities, and combined home visits and health facilities. Such interventions can be replicated in the SSA context to improve stimulation practices.

### Study limitations

Despite the longitudinal nature of our study, we could not confidently draw a conclusive link between parental stress, parenting practices, and child development based on these findings. The frequency of practice reported in this study was limited to one week. Noting that the data from this study came from two distinct trials with different study designs, it might be difficult to separate naturally occurring effects and effects related to the intervention. Another limitation of this study is that the data on parental stress and stimulation practices were self-reported, which could have introduced reporting biases, such as social desirability. In addition, although the study utilised datasets from two different settings, these findings may only be generalised to populations with similar characteristics. This, therefore, makes these findings indicative of such associations but does not prove that they are causal.

Future studies should focus on the frequencies of stimulation activities and real-time measurements of the effects of parental stress on stimulation activities and child developmental outcomes. The utilisation of Ecological Momentary Assessment (EMA) methods coupled with technology (wearable sensors such as actigraphy sensors) in the SSA setting could generate further evidence of the associations between PSS, child stimulation practices, and children’s developmental outcomes [56].

### Conclusion and policy implications

This study examined the associations between parental stress, parental stimulation activities, and child developmental outcomes among caregivers in low-resource SSA settings. The findings consistently showed a significant positive association between stimulation practices and children’s developmental outcomes at all the study sites. However, the association between parental stress, stimulation practices, and child developmental outcomes was not universally supported across the two study sites. The findings of this study, therefore, contribute to the evidence of the associations between PSS, parenting/child stimulation practices, and child developmental outcomes. Due to the significance of early stimulation to the child’s optimal growth and development, these findings highlight the need for policies and interventions aimed at preventing or reducing parental stress and boosting or enhancing child stimulation practices. This can be achieved by addressing poverty, low parental education, and other factors that underlie parental stress.

## Data Availability

The datasets used and analysed during the current study are available from the corresponding author upon reasonable request.

## Abbreviations

ASQ: Ages and Stages Questionnaire
ECD: Early Childhood Development
ESRC: Ethics and Scientific Review Committee
IRB: Institutional Review Board
PSS: Parental Stress Score
SSA: Sub-Saharan Africa
